# Hodgkin’s Disease

**Published:** 1918-05

**Authors:** 


### ABSTRACTS

**Hodgkin's Disease.** C. C. Simmons and Geo. Benet (sic), Boston M. & S. Jour., Dec. 13, 1917, report on cases seen at the Huntington Memorial Hospital, Apr. 1913-July 1916. 31 cases clinically diagnosed, applied for treatment. 3 proved to be lympho-sarcoma and 1 metastatic carcinoma, in 5 no specimen was obtained and 3 were examined in other hospitals and the data were considered inadequate. Thus 19 cases were established. The diphtheroid bacillus described by Bunting and Yates has been found in most cases examined by many authorities. Rosenow found it in 38 of 40 cases. The authors report positive findings in 2 out of 3 cases examined (Ordway). Inoculation experiments produced tuberculosis in a monkey from 1 case, resulted negatively in a monkey in another, negatively in a monkey and guinea pigs in another, negatively in guinea pigs in another. A portion of a gland removed for examination was re-implanted under the skin of the same patient, remained palpable for several days and disappeared (Tyzzer).

Incidence by sex was 6 females to 13 males, corresponding approximately to Zeigler's 71 females to 149 males, total 220. The distribution by decades, from 10 years upward was, respectively: 6, 8, 3, 2, (40-50). The duration, from the first symptom noted till death averaged 18 months as against 30 months for the three lympho-sarcoma cases. 6 cases lasted less than a year; 4 1-2 years; 2 2-3 years; 1 case 5 years and 1 8 years.

The blood counts were quite variable, though the relationship to stage of the disease is not very well shown. The reds varied from nearly 6½ million in one case and from more than 5 million in several others, down to about half the normal. The whites, from 35,000 to 1,000. The haemoglobin corresponded pretty well to the red count, often somewhat low relatively. Polymorphic cells were mostly in the range of 80% and upward to 90% but were only 52 and 57% respectively in two counts. Temporarily good results are reported from X-ray and radium but 5 cases survive being rated: I in poor condition, 2 each in fair and good condition.

